# Plasma ceramides independently predict all-cause mortality in men aged 85^+^

**DOI:** 10.1093/ageing/afac136

**Published:** 2022-06-18

**Authors:** Timo E Strandberg, Mika Kivimäki, Annele Urtamo, Satu Jyväkorpi, Reijo Laaksonen

**Affiliations:** Clinicum, Faculty of Medicine, University of Helsinki, Helsinki, Finland; Helsinki University Hospital, Department of Medicine and Rehabilitation, Helsinki, Finland; University of Oulu, Center for Life Course Health Research, Oulu, Finland; Clinicum, Faculty of Medicine, University of Helsinki, Helsinki, Finland; Department of Epidemiology and Public Health, University College London, London, UK; Clinicum, Faculty of Medicine, University of Helsinki, Helsinki, Finland; Clinicum, Faculty of Medicine, University of Helsinki, Helsinki, Finland; Finnish Cardiovascular Research Center, University of Tampere, Tampere University Hospital, Tampere, Finland; Zora Biosciences Oy, Espoo, Finland

**Keywords:** Ceramides, elderly, mortality, risk factor

## Abstract

**Background:**

assessing cardiovascular and mortality risk with conventional biomarkers is challenging in oldest-old due to multimorbidity and polypharmacy. Ceramides are bioactive lipids shown to predict mortality in late middle-aged cohorts.

**Objective:**

to assess whether plasma ceramides have independent prognostic value for mortality among oldest-old (85+).

**Design:**

longitudinal cohort study (Helsinki Businessmen Study, HBS) with a 3.5-year follow-up.

**Setting and subjects:**

survivors of HBS (125 men born in 1919–1934) visited the clinic for laboratory and clinical examination.

**Methods:**

functional status including physical (short physical performance battery) and Montreal Cognitive Assessment (MoCA) cognitive performance was assessed and laboratory examinations included a large set of biomarkers. Plasma ceramide concentration (Cer(d18:1/16:0)) was measured using a targeted liquid chromatography–tandem mass spectrometry assay. Mortality was retrieved from national registers.

**Results:**

median age was 88 years, two-thirds had multimorbidity and 59% were on statin treatment. During the follow-up, 22 (18%) men died. In a model adjusted for variables associated with mortality in the whole cohort at *P* < 0.20 (log glucose, SPPB, MoCA and statin use), Cer(d18:1/16:0) as a continuous trait was associated with increased mortality: hazard ratio (HR) per 1 SD 1.64 (95% confidence interval [CI] 1.23–2.18). Compared with the bottom tertile of Cer(d18:1/16:0), HR of mortality was 5.44-fold (95% CI 1.17–25.3) in the top tertile.

**Conclusions:**

these data raise the hypothesis that plasma ceramide concentrations and especially Cer(d18:1/1:60) may offer a clinically useful biomarker to evaluate prognosis in very old age. Such biomarkers are needed for geriatrics, where multimorbidity and pharmacotherapies, such as statins are prevalent hampering assessment of prognosis using conventional methods.

## Introduction

Dyslipidaemia is an important risk factor for cardiovascular disease and all-cause mortality in the general population. The role of dyslipidaemia as a risk factor is more obscure among oldest-old—over 85 years of age—due to frailty, subclinical conditions, multimorbidity and polypharmacy including frequent statin use [1]. Overall, in this oldest age group, common risk prediction scores are not helpful for stratifying cardiovascular risk, because almost all individuals aged ≥70 years exceed conventional risk thresholds [2]. The Framingham risk score, for example, only applies to those aged 30–79 years [3]. Consequently, assessing cardiovascular and mortality risk in oldest-old patients is difficult complicating safe decisions about deprescribing cardiovascular medications.

This implicates an unmet need for reliable predictors as the number of oldest-old individuals is rapidly growing. Among people reaching high age, the number of years to live may still be substantial. For example, an 85-year-old man can have a 75% chance of surviving 2 years and a 25% chance of living 9 years. Although heart disease coronary calcium score may be useful for the assessment risk even in older patients [4], reliable, scalable and easy-to-assess blood-based biomarkers are additionally needed.

Ceramides are bioactive molecular lipids, which have a role in several cellular functions and implicated in apoptosis, inflammation, obesity and insulin resistance, and are present in small amounts in plasma [5]. Measurement of plasma concentrations of ceramides has been found to improve cardiovascular and all-cause mortality risk prediction over other variables like low-density lipoprotein (LDL)-cholesterol in midlife [6–8], but whether this applies to oldest-old individuals is unknown. We examined this in the oldest home-dwelling survivors (median age 88 years) of a longitudinal study.

## Methods

This is a cohort study of a socioeconomically homogenous group of Caucasian men born in 1919–1934 (original *n* = 3,490) [9]. They have been followed-up since midlife from the 1960s with regular questionnaire surveys from 2000 onwards (response rates between 65 and 82%). In 2017, a convenience subcohort of home-dwelling and functionally independent survivors (*n* = 130) participated in clinical investigations including questionnaires about lifestyle and health-related quality of life, chronic diseases and medications, and tests of psychological (Montreal Cognitive Assessment, MoCA) and physical function (short physical performance battery, SPPB). A wide set of laboratory tests, including plasma lipids, after a 12-h fast were analysed in the routine laboratory of the University Hospital. Four circulating ceramides, Cer(d18:1/16:0), Cer(d18:1/18:0), Cer(d18:1/24:0) and Cer(d18:1/24:1) in plasma samples (*n* = 125) were quantified using a targeted liquid chromatography–tandem mass spectrometry assay, and the Coronary Event Risk Test (CERT) was calculated as previously described [6]. Laboratory personnel were blinded to the characteristics of the cohort. All-cause mortality through January 2021 was retrieved from the national Population Information system with a follow-up of 3.5 years.

We used receiver operating curve analysis to compare the area under the curve (AUC) of various ceramides for all-cause mortality. Preliminary analyses showed that of various ceramides measured, Cer(d18:1/16:0) was the best predictor in this cohort (AUC 0.71, 95% CI 0.58–0.80).

Cer(d18:1/16:0) concentration was divided into tertiles and various clinical and laboratory variables were compared with analysis of covariance. After confirming that proportional hazards assumption was not violated (Schoenfeld residuals), we used Cox proportional-hazards models (hazard ratios [HR] with 95% confidence interval [CI]) to investigate Cer(d18:1/16:0) as a predictor of all-cause mortality. Cer(d18:1/16:0) was used both as a continuous variable and as divided into tertiles (lowest tertile as the reference).

In addition to adjusting for age only, three multivariably adjusted models were used to analyse the independent role of Cer(d18:1/16:0): **Model 1** was adjusted for variables (other than ceramides) associated with mortality in the whole cohort at *P* < 0.20 (log glucose, SPPB, MoCA and statin use); **Model 2** was adjusted for factors used in the Framingham equation (age, smoking, cholesterol, high-density lipoprotein (HDL) cholesterol, systolic blood pressure); **Model 3** was adjusted for factors which were different between Cer(d18:1/16:0) tertiles at *P* < 0.20 (total, HDL- and LDL-cholesterol, triglycerides, log C-reactive protein, albumin and statin use). For comparison, we also tested the effect of substituting Cer(d18:1/16:0) with LDL-cholesterol in Model 1. To illustrate these associations, we plotted Kaplan–Meier survival curves comparing tertiles of LDL-cholesterol and Cer(d18:1/16:0). NCSS 2020 statistical software (ncss.com) was used for statistical analyses. *P*-values <.05 were taken as statistically significant.

## Results

At baseline, median age (interquartile range) of the 125 men was 88 (86–90) years, mean (SD) body mass index of 25.8 (2.8) kg/m^2^. Medians (interquartile range) for important functional characteristics in older people, the SPPB, MoCa and walking speed were 10 points (8–11), 24 points (22–25) and 0.9 m/s (0.8–1.1), respectively. Two-thirds had multimorbidity and 59, 51.9 and 15.2% were on statin, antihypertensive and antidiabetic drug treatment, respectively. Only two men used insulin and no one was using proprotein convertase subtilisin/kexin type 9 (PCSK9) inhibitors or ezetimibe.

Mean LDL-cholesterol was 2.5 (SD 0.9) mmol/L. Correlation between Cer(d18:1/16:0) and LDL-cholesterol was significant but not very strong (Spearman r = 0.63; *P* < 0.001), and concentration of Cer(d18:1/16:0) was significantly lower among statin users (mean 2.49 μmol/L×10, SD 0.51) than nonusers (mean 2.96, SD 0.67; *P* < 0.001). Clinical and laboratory data of the men by Cer(d18:1/16:0) tertiles are shown in Table 1. Between 2000 and 2017, statin use increased from 17.5 to 84.2% in the bottom, from 14.0 to 58.1 in the intermediate and from 7.0 to 39.5% in the top tertile. No differences between the tertiles were observed in the use of antihypertensive or antidiabetic medications.

**Table 1:** Participant characteristics according to plasma ceramide Cer(d18:1/16:0) tertiles

Variable	All, *n* = 125	Cer(d18:1/16:0), bottom tertile, *n* = 40	Cer(d18:1/16:0), intermediate tertile, *n* = 43	Cer(d18:1/16:0), top tertile, *n* = 42	*P* value between groups
Cer(d18:1/16:0), μmol/L×10	2.67 (0.62)	2.06 (0.17)	2.57 (0.16)	3.34 (0.52)	<0.001
CERT score, points	5.1 (3.1)	3.2 (2.2)	4.4 (2.2)	7.5 (2.9)	<0.001
-median (IQ range)	5 (3–7)	3 (1–5)	5 (2–6)	8 (5–10)	
Age, median (IQ range)	88 (86–90)	88 (86–90)	88 (86–90)	88 (86–90)	0.31
BMI, kg/m^2^	25.8 (2.8)	26.4 (2.7)	25.6 (2.4)	25.5 (3.1)	0.29
Smoker, *n* (%)	2 (1.6)	0	1 (2.3)	1 (2.3)	0.81
Ever smoker, *n* (%)	58 (46.0)	16 (40.0)	21 (48.9)	21 (48.9)	0.21
Systolic blood pressure, mmHg	140 (3)	138 (3)	138 (3)	145 (3)	0.27
Diastolic blood pressure, mmHg	82 (2)	81 (2)	81 (2)	85 (2)	0.27
Walk speed, median (IQ range), m/s	0.9 (0.8–1.1)	0.9 (0.8–1.0)	0.9 (0.7–1.1)	1.0 (0.8–1.1)	0.45
SPPB, median (IQ range), points	10 (8–11)	10 (8–11)	10 (8–11)	10 (8–12)	0.85
MoCA, median (IQ range), points	24 (22–25)	24 (22–25)	24 (22–25)	23 (22–25)	0.50
Plasma lipids, mmol/L					
-Total chol, mmol/L	4.4 (0.8)	3.8 (0.7)	4.3 (0.8)	5.2 (1.0)	<0.001
-HDL-chol	1.5 (0.3)	1.4 (0.3)	1.4 (0.4)	1.6 (0.4)	0.07
-LDL-chol	2.5 (0.9)	2.0 (0.6)	2.4 (0.7)	3.2 (0.9)	<0.001
-Triglycerides	1.1 (0.4)	1.0 (0.3)	1.2 (0.4)	1.2 (0.4)	0.01
Glucose, mmol/L	6.2 (1.0)	6.1 (0.7)	6.4 (1.3)	6.3 (0.9	0.24
Insulin, mU/L	7.7 (4.7)	8.4 (5.0)	8.1 (4.6)	6.8 (4.7)	0.24
C-reactive protein, median (IQ range) mg/L	1.4 (0.8–3.1)	1.0 (0.6–1.5)	1.8 (0.9–4.4)	1.5 (0.9–3.4)	0.009 (log)
Albumin, g/L	37.8 (3.1)	38.4 (2.7)	37.1 (3.5)	37.7 (2.9)	0.19
ALT, U/L	24.3 (26.1)	22 (15–30)	19 (15–26)	21 (16–27)	0.70
Creatinine, median (IQ range)	96 (82–114)	92 (81–110)	99 (79–116)	91 (82–116)	0.58
BNP, median (IQ range), ng/L	103 (53–223)	96 (44–211)	100 (50–232)	110 (62–180)	0.95
Multimorbidity^a^, *n* (%)	80 (63.6)	28 (69.2)	27 (63.4)	25 (59.9)	0.59
Statin use, *n* (%)	74 (59.2)	32 (84.2)	25 (58.1)	17 (39.5)	<0.001
Antihypertensive use, *n* (%)	64 (51.9)	20 (50.0)	21 (48.9)	23 (54.7)	0.90
Antidiabetic drug use, *n* (%)	19 (15.2)	9 (22.5)	5 (11.6)	5 (11.9)	0.28
Mortality by 2021	22 (17.6)	2 (5)	7 (16.3)	13 (31.0)	0.008

Continuous values are mean (SD) unless otherwise indicated. ALT, alanine aminotransferase; BMI, body mass index; BNP, brain natriuretic peptide; CERT, The Coronary Event Risk Test; IQ, interquartile range; MoCA, Montreal Cognitive Assessment; SPPB, Short Physical Performance Battery.

^a^Multimorbidity: ≥2 of the following: diabetes, coronary artery disease, heart failure, peripheral artery disease, stroke, chronic pulmonary disease, cancer, musculoskeletal disease.

During the 3.5-year follow-up, 22 men (18%) died. In an age-adjusted Cox regression analyses, higher Cer(d18:1/16:0) as a continuous variable was associated with increased mortality: HR per 1 SD was 1.69 (95% CI 1.25–2.30). The HR remained unchanged in Model 1: 1.64 (95% CI 1.23–2.18) and was higher in Model 2 (2.78 [1.49–5.11]) and Model 3 (2.71 [1.45–5.07]), which both included total cholesterol and LDL-cholesterol. Adding multimorbidity and use of antihypertensives to Model 1 did not essentially change the result (HR 1.83, 95% CI 1.31–2.54).

In analysis in which Cer(d18:1/16:0) was replaced with continuous LDL-cholesterol, HR per SD higher LDL-cholesterol was non-significant (1.20; 95% CI 0.77–1.86; Model 1). This difference between Cer(d18:1/16:0) and LDL-cholesterol as a predictor of mortality is illustrated in the figure showing overall survival curves by Cer(d18:1/16:0) tertiles (log-rank *P* = 0.01) and LDL-cholesterol tertiles (log rank *P* = 0.71). The results of Cer(d18:1/16:0) tertiles were consistent with those obtained using plasma Cer(d18:1/16:0) as a continuous variable. In Model 1 and compared with the bottom tertile, HR for mortality was 2.53-fold (95% CI 0.52–12.3) and 5.44-fold (95% CI 1.17–25.3) for the intermediate and top tertile of Cer(d18:1/16:0), respectively.

**Figure 1:**
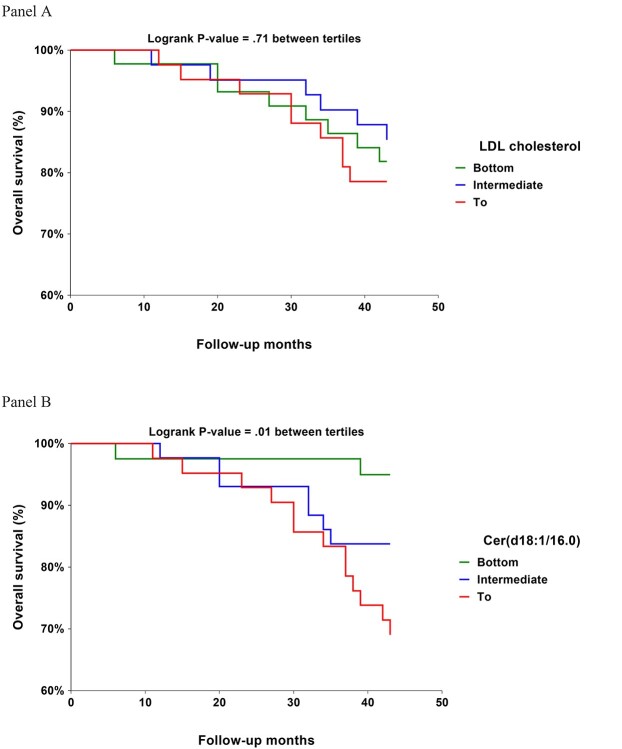
Overall survival curves by tertiles of LDL cholesterol (Panel A) and Cer(d18:1/16:0) (Panel B) during 3.5-year follow-up among 125 home-dwelling men with median age of 88 years at baseline.

## Discussion

Although valuable in midlife, prognostic value of common risk indicators, such as LDL-cholesterol is weak or non-existent in old age. In our cohort of community-living oldest-old men, plasma ceramide level, specifically Cer(d18:1/16:0) predicted all-cause deaths independently of common laboratory, cognitive and physical function variables, including SPPB and LDL-cholesterol, during a 3.5-year follow-up. The predictive value was also independent of multimorbidity, statin or antihypertensive use, all very prevalent in this older cohort. To the best of our knowledge, there are no previous studies about ceramides specifically in the oldest-old.

### Strengths and limitations

Due to the observational design, causal relationships cannot be established. Further limitations include the relatively small number of survivors in a follow-up study of a socioeconomically selected male cohort. Despite the small number, the results were statistically significant and biologically plausible in light of studies in younger and late middle-aged people. Cause of death was not established, but cardiovascular disease, clinical or subclinical, is very common among people aged 80 years and over which may explain why a lipid biomarker, such as ceramides predicted all-cause mortality. However, in contrast to ceramides, LDL-cholesterol, an established predictor of cardiovascular disease, did not consistently predict mortality in the present cohort of oldest-old (Figure 1). This difference may be explained by observations that unlike LDL-cholesterol, ceramides also predict non-cardiovascular mortality among individuals aged <75 [10].

### Previous studies

In agreement with our findings, the value of ceramides as an independent prognostic indicator has been implicated in several studies consisting mostly of 60-to 70-year old people [5–8]. Although the evidence-base is growing, routine use of ceramides has not yet been established at the guideline level, such as the 2021 European guidelines on cardiovascular disease prevention in clinical practice [11].

### Clinical implications

When a new prognosis marker is suggested in medicine, the following points must be considered: (i) Does it give added value to existing and established risk factors, (ii) Is the factor clinically feasible to measure and what is its cost, (iii) Are there existing methods to modify the risk factor if it is causal. We argue that ceramides may fulfil all these points. Point 1 has been indicated in younger cohorts and the present results extend this knowledge to oldest-old. According to our results, ceramides appear to be especially useful for excluding risk: using the lowest tertile as cutpoint, the negative predictive value was as high as 95%. As to point 2, ceramide measurement is generally available to clinicians both in Europe and the US at a reasonable cost (<30 to 50 euros/USD). Finally, ceramide concentrations can be lowered by current therapies such as dietary or statin and PCSK9 inhibitor medications [5, 12]. A special advantage for ceramides in geriatrics could be to guide decisions for primary prevention and possibly for monitoring effects of deprescribing of statins or other preventive medications. These aspects should be tested in appropriate future trials.

## Conclusions

Our findings suggest that plasma ceramide concentrations and especially Cer(d18:1/16:0) may offer a clinically useful and independent biomarker to evaluate overall prognosis in very old age. Such biomarkers are needed in geriatrics, where multimorbidity and, for example, statin and antihypertensive medications are prevalent hampering assessment of prognosis using conventional methods. The results from this socioeconomically selected, and relatively well-functioning cohort are hypothesis-generating and must be verified in larger datasets.
